# Fish Community Resource Utilization Reveals Benthic–Pelagic Trophic Coupling Along Depth Gradients in the Beibu Gulf, South China Sea

**DOI:** 10.3390/biology14020207

**Published:** 2025-02-16

**Authors:** Xiaodong Yang, Konglan Luo, Jiawei Fu, Bin Kang, Xiongbo He, Yunrong Yan

**Affiliations:** 1 Fisheries College, Guangdong Ocean University, Zhanjiang 524088, China; yangxd2832@163.com (X.Y.); luokonlan@gmail.com (K.L.); fjw18884902029@outlook.com (J.F.); 2 Fisheries College, Ocean University of China, Qingdao 266003, China; kangbin@ouc.edu.cn; 3 Guangdong Provincial Engineering and Technology Research Center of Far Sea Fisheries Management and Fishing of South China Sea, Guangdong Ocean University, Zhanjiang 524088, China

**Keywords:** marine food webs, benthic–pelagic coupling, Bayesian mixing models, environmental gradients, adaptive foraging

## Abstract

Benthic–pelagic coupling is a key approach to studying the structure and energy dynamics of shallow marine food webs. The movement and foraging patterns of consumers are major drivers of nutrient and energy distribution in ecosystems and are critical for maintaining ecosystem stability. To better understand the energy coupling of consumers between coastal marine habitats, this study employed a Bayesian mixture model using SC and SI data. By classifying functional groups based on taxonomy, morphological traits, and feeding ecology similarities, we constructed a trophic network and analyzed the changes in fish feeding patterns and the dynamics of benthic–pelagic coupling across environmental gradients. The results show that the primary carbon sources in the Beibu Gulf are phytoplankton, particulate organic matter (POM), and sediment organic matter (SOM), with phytoplankton contributing the most. Pelagic food subsidies dominate the food web. Small sized, abundant planktivorous and benthivorous fish act both as predators and important prey, transferring carbon and energy derived from both benthic and pelagic zones to higher trophic-levels. Larger, higher-trophic-level piscivorous fish serve as key energy couplers, preying on organisms from various habitats. Depth and chlorophyll–a (Chl–a) are the two key variables influencing the trophic structure of fish, with opposite gradient patterns observed for each. Along the depth gradient, fish exhibit clear adaptive foraging strategies. As water depth increases, fish tend to forage more within their specific habitat (either benthic or pelagic), with prey types continually changing, leading to a gradual reduction in the strength of benthic–pelagic trophic coupling. This study reveals the spatial resource utilization patterns and adaptive foraging strategies of fish in the Beibu Gulf, providing deeper insights into the structure and spatial variation of food webs. It also enhances our understanding of ecosystem responses to human pressures and global changes, offering valuable perspectives for predicting these responses.

## Simple Summary

This study analyzed the biological and physical factors driving trophic variability in fish feeding using Generalized Additive Models (GAMs). Bayesian mixing models based on stomach content (SC) and stable isotope (SI) data were employed to investigate the trophic network structure associated with fish and explore the dynamic changes in benthic–pelagic coupling mediated by fish predation across environmental gradients. The findings reveal the spatial resource utilization patterns of fish in the Beibu Gulf, deepen our understanding of energy coupling dynamics between different habitats, and provide important theoretical insights into the mechanisms underlying the formation of the local food web structure.

## 1. Introduction

The food web represents the processes of energy processing and transformation, encompassing information about species composition and trophic interactions [1], thus reflecting the fluxes of energy and matter within ecosystems. Estuarine and coastal ecosystems (ECEs), typically characterized by shallow waters, are minimally influenced by the ocean, highly impacted by coastal runoff, and frequently subjected to external pressures (such as climate change or anthropogenic activities). These factors contribute to the complexity of nutrient inputs and energy transfer within ECE food webs while also enhancing the coupling of different energy pathways [2,3].

Understanding how energy and matter flow between different habitats in a food web is a central issue in ecology [4]. Investigating the food web structure and energy dynamics in shallow marine ecosystems through benthic–pelagic coupling is an effective approach [5,6,7,8,9,10]. The benthic–pelagic coupling process includes both passive processes (e.g., sedimentation) and active processes (e.g., predation and other biological processes) [11], representing the integration of resources by consumers from different habitats [12,13,14,15]. For example, organic matter derived from the pelagic zone may sink to the seafloor or plankton may migrate vertically, becoming food for benthic organisms [11], or benthic organic matter can be consumed by consumers and transferred up the food chain to the pelagic zone [16]. Coupling processes have been shown to be influenced by environmental variables, such as primary production intensity [17,18,19,20,21,22] and water depth [8,23,24], and are also directly and indirectly regulated by biological factors (e.g., species composition, community structure, and functional traits), which drive changes in food web structure and ecosystem functioning [25]. Depth, temperature, and other environmental variables are key factors influencing changes in biological composition [26,27]. Therefore, exploring the dynamics of benthic–pelagic coupling along environmental gradients is crucial for understanding the spatial variation in marine food web structure and predicting ecosystem responses to anthropogenic pressures or global change.

In the Beibu Gulf, nektonic benthos such as large zooplankton (e.g., Sergestidae, Mysidacea) and small fish species play a critical role as key carriers of coupled benthic and pelagic food webs [28]. Fish with strong migratory adaptability, including both horizontal and vertical migration, can break through the physical barriers between the benthic and pelagic zones, thus serving as important connectors in the coupling of benthic and pelagic ecosystems [15,24]. In the spatially and temporally heterogeneous environments of the region, fish typically adapt to varying habitats through foraging strategies [29,30]. The flexibility of these foraging strategies is generally influenced by factors such as the fish’s foraging behavior (e.g., habitat preference, morphological traits, and mobility) [31,32,33,34], individual traits (e.g., digestive organ characteristics, ontogenetic shifts in feeding) [35,36,37], and the abundance and types of prey available [38,39]. The pelagic fish species, such as *Decapterus maruadsi*, *Trachurus japonicus*, and *Psenopsis anomala*, have small or reduced teeth on both their upper and lower jaws, and their gill rakers are fine and well developed, enabling them to primarily filter plankton for food [40]. However, the Beibu Gulf is also rich in small forage fish and shrimp [41,42], which leads us to hypothesize that, under certain environmental conditions, larger pelagic fish species that primarily feed on zooplankton may switch to preying on these small fish and shrimp in abundance. The flatfish (Pleuronectiformes), characterized by a laterally flattened, oval, or tongue-shaped body, and asymmetry, typically reside on the seafloor and feed on benthic organisms. However, due to the high abundance of foundational resources in the shallow waters of the Beibu Gulf (such as juvenile fish and large zooplankton like Sergestidae), these resources are easily vertically displaced to the benthic zone and are likely to be consumed by flatfish in large quantities. Additionally, some dominant fish species in the region, such as *Saurida*, *Trichiurus*, and *Pennahia*, are fierce predators with strong, sharp jaws and large oral fissures. These species primarily prey on small- and medium-sized fish [37,43]. As apex predators in the food chain [44], their foraging strategies tend to be opportunistic. We expect that in deeper waters or areas with high benthic prey abundance, these fish are more likely to target benthic organisms. Therefore, changes in the structure of fish and prey communities across different environmental gradients in the Beibu Gulf may alter the dynamics of the food web and affect the coupling pathways between the benthic and pelagic zones. However, the effects of fish predation on the coupling patterns between benthic and pelagic zones, as well as their relative importance, are still poorly understood. This coupling effect is crucial for understanding the dynamic structure and function of marine food webs.

**Figure 1:**
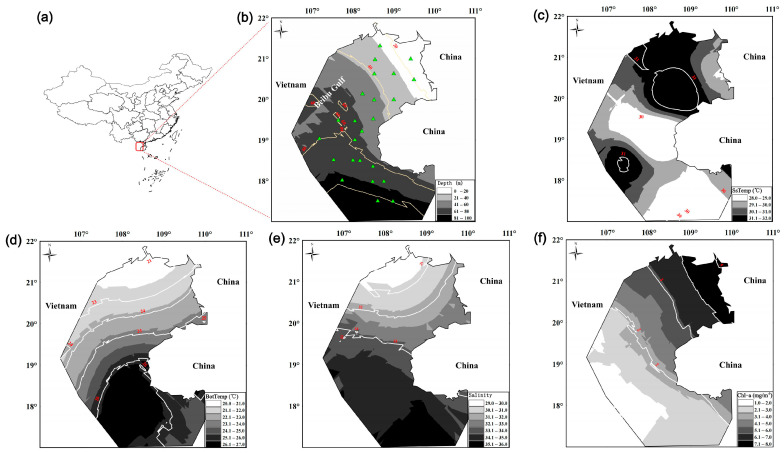
Spatial characteristics of sampling in the Beibu Gulf. (**a**,**b**) Sampling station maps, with green triangles indicating the stations; (**b**–**f**) Kriging interpolation of biological and environmental variables, including water depth (depth), surface temperature (SsTemp), bottom temperature (BotTemp), chlorophyll a concentration (Chl–a), and salinity. The white solid lines are the isoclines.

The Beibu Gulf, located in the northwest of the South China Sea (SCS), is a semi-enclosed bay (Figure 1). The coastline is characterized by diverse ecosystems, including estuaries, bays, coral reefs, and mangrove wetlands, with a recorded fish species diversity of 1059 species [45]. The Northern Gulf is primarily influenced by coastal currents (CCs) and the West Guangdong coastal current (WGCC), while the Southern Gulf is mainly affected by the intrusion of high-salinity, high-temperature South China Sea water (SCSW). As a result, from north to south, there is a distinct environmental gradient with increasing water depth, which affects parameters such as water temperature, salinity, and Chl–a [46,47]. These gradients lead to significant spatial variation in primary productivity, planktonic communities, and the composition and abundance of benthic organisms [48,49,50]. For fish with high mobility, the spatial variability in the availability of prey resources (such as nektonic benthos) may lead to spatial changes in the coupling strength between benthic and pelagic food webs. Seasonal or spatial replacement of prey may further result in differences in the coupling intensity between benthic and pelagic pathways, ultimately influencing the dynamics of energy flow and trophic relationships within the food web.

This study aims to investigate the role of fish-dominated biological communities in the energy coupling between benthic and pelagic habitats within the food web of the Beibu Gulf, and the spatial variation in this role. Given the strong mobility, adaptive foraging, and high biodiversity of fish in the Beibu Gulf, we hypothesize that fish are key connectors in the benthic–pelagic coupling. Furthermore, we propose that depth and primary production intensity gradients, from north to south and from coastal to offshore regions, will significantly influence changes in fish feeding strategies, thereby driving alterations in the strength of benthic–pelagic coupling. To this end, this study uses stomach content (SC) and stable isotope (SI) data (carbon isotope (δ^13^C) and nitrogen isotope (δ^15^N)), and applies Bayesian mixing models, such as SIMMr [51] and MixSIAR [19], to address the following objectives: (1) determine the trophic network structure associated with fish; (2) identify the biological and physical drivers of spatial variability in fish feeding (trophic variation); and (3) explore changes in resource utilization by fish communities along the gradients of these drivers.

## 2. Materials and Methods

### 2.1. Data Collection

#### 2.1.1. Sample Collection

Biological and environmental data from the Beibu Gulf (105.67–110.17° E, 17–21.75° N) were collected during fishery surveys in August 2022. A total of 24 sampling stations were established based on depth gradients (Figure 1a,b). Macroalgae, invertebrates, and fish data were sampled at each site using a 441 kW vessel and a bottom trawl with a 38.5 m corkline length and a 20 mm cod-end mesh. At each site, the trawl was towed for 1 h at a speed of 3 knots. The samples were identified to the species level whenever possible, with 3 to 60 individuals sampled per fish species at each station. A small number of macroalgae, which were mixed in with the catch, were selected, washed with distilled water, desalinated, and then wrapped in aluminum foil at each station. Particulate organic matter (POM) and phytoplankton samples were collected from the surface waters using a 5L Niskin sampler at 0.5 m below the surface. The water samples were pre-filtered through a 200 μm mesh to remove large inorganic particles and zooplankton. A portion of the pre-filtered water was then passed through a pre-combusted GF/F filter (heated at 450 °C for 4 h to remove organic matter) to obtain POM samples. Another portion of the water was allowed to settle for 24 h, and the supernatant was siphoned and filtered through a pre-combusted GF/F filter to obtain phytoplankton samples. Zooplankton were vertically collected using a 180 μm mesh plankton net. Sediment samples were collected using a 1/16 m^2^ Petersen grab from approximately 1 cm above the sediment surface to obtain sediment organic matter (SOM) samples. All samples were frozen at −20 °C until laboratory analysis.

In addition, explanatory variables that may influence the trophic variation in the Beibu Gulf were also collected, including water depth (depth), surface temperature (SsTemp), bottom temperature (BotTemp), surface salinity (salinity), and chlorophyll–a (Chl–a) (Figure 1b–f). A 1L water sample for Chl–a measurement was pre-filtered through a 200 μm mesh sieve and then frozen in liquid nitrogen for transport to the laboratory. The sample was extracted for 20 h using a 93% acetone solution. The extract was analyzed on a fluorometer (Turner Designs, Sunnyvale, California, USA), using the non-acidic module and calibrated with Sigma chlorophyll standards (C6144) to determine the Chl–a. Other environmental variable data were obtained from in situ CTD measurements (SBE19PLUS).

#### 2.1.2. Stable Isotope Analysis

Fish samples were measured for total length (cm), and a sample of white dorsal muscle tissue was dissected. For invertebrates, tissue samples were taken from the muscle of crustaceans, the adductor muscle of bivalve mollusks, and the mantle of cephalopods. In addition, fish samples were dissected to observe and record the types of stomach contents. The stomach contents were identified to the lowest possible taxonomic level based on the morphological characteristics of the remains.

POM and phytoplankton were acidified by exposure to hydrochloric acid fumes for 24 h to remove inorganic carbon, followed by rinsing with Milli–Q water (ZIQ7000T0C, Kenilworth, New Jersey, USA). SOM samples were treated with an excess of 1 mol/L hydrochloric acid solution, and the mixture was stirred until the reaction was complete (until no bubbles were produced, indicating the removal of inorganic carbon), then rinsed with Milli–Q water until it achieved a neutral pH (pH = 7) [52]. From the zooplankton samples, copepods and macro-zooplankton, such as juvenile fish and crustacean larvae, were selected and rinsed with Milli–Q water. All specimens were then subjected to freeze-drying at −55 °C for 48 h using a freeze-dryer (Alpha1–4/2–4LD Plus, Christ, Osterode, Germany). After freeze-drying, the specimens were homogenized into a powder using a steel bead homogenizer (MiniBeadbeater–16, Biospec, Bangor, PA, USA). Approximately 0.40 mg of powder from each specimen was weighed on a Mettler Toledo microscale and packed. The specimens were analyzed using an elemental analyzer (EA IsoLink, Thermo Fisher Scientific, Waltham, MA, USA) connected to an isotope ratio mass spectrometer (Thermo Scientific 253 Plus, Thermo Fisher Scientific, Waltham, MA, USA) at Guangdong Ocean University, China. All stable isotope values are reported in δ notation.

(1) $$\delta X=\left(\frac{R_{\mathrm{sample}}}{R_{\mathrm{standard}}}-1\right)\times 10^{3}$$

where R represents the ^13^C/^12^C or ^15^N/^14^N ratios, with standards being atmospheric nitrogen and Pee Dee Belemnite (PDB). Using the elemental carbon to nitrogen ratio (C:N) as a proxy for lipid content, the mathematical correction for lipids proposed by Post et al. [53] was applied to all samples with C:N ratios higher than 3.5:

(2) $$\delta^{13}C_{\mathrm{corrected}}=\delta^{13}C_{\mathrm{uncorrected}}-3.32+0.99\times C:N$$

#### 2.1.3. Correction of Isotopic Values for Spatial Variation

The δ^15^N and δ^13^C values of species provide information on trophic levels (TLs) and energy pathways relative to the trophic baseline, while muscle tissue integrates isotopic signatures that represent an individual’s diet over the past few months. Since the isotopic ratios of primary producers vary greatly over time, and primary consumers, compared to primary producers (e.g., phytoplankton), have the advantage of buffering short-term fluctuations (such as seasonal changes, environmental factors, or other short-term variations) in isotopic values [54], this study selected filter-feeding bivalves [55,56], such as *Aequipecten opercularis*, as the trophic baseline, as they rely on a mixture of food sources from both benthic and pelagic zones [57].

At the same time, considering that the isotopic values of the trophic baseline and higher-trophic-level species may change spatially due to environmental gradients, specifically along the nearshore-to-offshore gradient, the influence of terrestrial nutrients and detritus gradually decreases, and the δ^15^N and δ^13^C ratios of these terrestrial sources differ from those of marine substances. Therefore, we corrected the nitrogen and carbon isotopic values of all consumers based on the spatial variation in baseline values (Figure S1). Any SI data mentioned in this study refer to baseline-corrected SI values. The nitrogen and carbon isotopic values for each consumer sample were corrected by subtracting the baseline value of the sampling location and adding the average baseline value for the entire study area:

(3) $$\delta X_{\mathrm{corrected}}=\delta X_{\mathrm{consumer}}-\delta X_{\mathrm{local}\;\mathrm{baseline}}+\delta X_{\mathrm{mean}\;\mathrm{baseline}}$$

### 2.2. Data Analysis

#### 2.2.1. Food Web Modeling

A total of 1319 samples were collected and analyzed from a depth range of 10 to 90 m, including primary carbon sources, zooplankton, large invertebrates, fish, and cephalopods, representing 139 trophic species with sample sizes ranging from 3 to 46. A trophic species refers to a group of organisms that share the same predators and prey (which may include one or more species) [58]. These trophic species typically correspond to taxonomic species, but may also include higher taxonomic groups. In this study, the term “species” refers to trophic species. Based on taxonomy (e.g., fish, cephalopods, crustaceans), feeding strategies (e.g., filter feeders, scavengers), morphological traits, digestive organ types, and feeding ecological types, the 139 species were qualitatively classified into 17 functional groups, ranging from primary producers to piscivorous fish and cephalopods (Table 1; Appendix A, Table A1). The stomach content data primarily come from over 10,000 measured stomach content statistics (Table S1).

The method proposed by Post [59] was used to estimate the trophic level of each species or taxonomic group:

(4) $${\mathrm{TL}}_{i}=\lambda_{\mathrm{base}}+\left[\left(\delta^{15}N_{i}-\delta^{15}N_{\mathrm{base}}\right)/\Delta^{15}N\right]$$

where λ_base_ is the corresponding base TL, assumed to be 2 in this case; δ^15^N_i_ is the δ^15^N value for individual i, and δ^15^N_base_ is the mean δ^15^N value for all *A. opercularis* samples. A trophic discrimination factor (TEF) (Δ^15^N) of 3.4 is used, representing the average ^15^N enrichment between the muscle tissue of fishes and invertebrates [59], with *A. opercularis* serving as the base trophic level. The trophic level of a species or taxonomic group is then calculated by averaging the TL of an individual (or species).

Based on SC analysis, a trophic link was established if at least one species in a functional group preyed upon a species in another functional group. A Bayesian mixing model (SIMMr package, R software version 4.1.11) [51] was used to estimate the relative contribution of each resource to consumer diets. The TEF must be considered in consumer metabolism, particularly the directional nitrogen TEF along TLs [60,61]. Therefore, the carbon TEF is set to Δ^13^C: (1.0 ± 0.4)%, and the nitrogen TEF is based on the proportionalized trophic framework proposed by Hussey [62], derived from experimental data and meta–analysis, rather than the conventional average nitrogen TEF of Δ^15^N = 3.4‰ (±1.0%). This method better reflects the actual TEF of the study area compared to those from the literature, significantly reducing uncertainty in the Bayesian model analysis. The proportionalized Δ^15^N for consumers is calculated using the following equation:

(5) $$\Delta^{15}N=5.92-0.27\times \delta^{15}N_{\mathrm{consumer}}$$

The nitrogen TEF (Mean ± SD) was obtained by averaging the Δ^15^N values of all species within a functional group. The model requires three types of input data: (1) the isotopic characteristics of the functional group (consumer); (2) the isotopic characteristics of food sources (functional groups), including means and standard deviations; (3) the TEF for the functional group (consumer). The model output provides the probability distribution of the relative contribution of each food source to each consumer’s diet, expressed as the mean ± standard deviation, along with the upper and lower limits of the credibility interval (CI 25%, 50%, 75%, 95%). For detailed information, see Supplementary Table S2.

#### 2.2.2. Isotopic Space in Relation to the Environment

Trophic interactions have been proposed as a bridge between biodiversity and ecosystem function [63,64]. Exploring the trophic interactions of fish within the food web and their variations along environmental gradients is crucial for understanding how marine food webs are constructed along environmental gradients, the drivers of food web structure, and predicting the response of coastal ecosystems to global change. To assess the isotopic space associated with the biophysical environment, we used multivariate Generalized Additive Models (GAMs) to examine the relationship between multiple environmental parameters and isotopic values, including δ^13^C and δ^15^N. Specifically, the GAMs were applied to predict isotopic values as a function of a combination of multiple parameters (e.g., Depth, SsTemp, BotTemp, Salinity, and Chl–a), rather than examining the effects of individual parameters separately. This approach allows for the modeling of complex, nonlinear relationships between isotopic values and several environmental factors simultaneously. The multivariate GAMs incorporate the effects of each predictor variable while accounting for potential interactions and smooth nonlinear trends. By using this method, we can better understand how combinations of environmental factors influence isotopic patterns. The models were implemented using the R package mgcv (version 1.8–31) [65].

At the functional group level, only groups related to fish were considered. The model selection process began with univariate models, progressively adding variables and running all possible combinations of explanatory variables. Given the complexity and difficulty in interpreting overly complex models, the models run had a maximum of four variables. During model selection, the model with the lowest Akaike Information Criterion for small sample sizes (AICc) was preferred [66], while improvements in R^2^ and the explained deviance percentage (Δ% dev) were also considered. Generalized cross-validation (GCV) was used to evaluate the predictive variables of the model; the smaller the value, the better the modeling ability. F-tests were used to evaluate the nonlinear contribution of nonparametric effects and assess the significance of each factor.

#### 2.2.3. Estimation of Predatory Fish Diet as a Function of Depth

To better infer the spatial impact of environmental conditions on fish functional groups, the Bayesian isotope mixing model MixSIAR [19] was used, based on the constructed food web (SIMMr model), to further explore the variation in fish trophic interactions along environmental gradients. The key predictor variables identified from the GAMs model were introduced as continuous covariates influencing the contribution of sources to the functional group’s diet. For each fish-related functional group, the MixSIAR model was run, maintaining the same trophic links, isotopic values for consumers and food sources and consumer TEF as those used in the SIMMr model. For each MixSIAR feeding estimate, the mean ± standard deviation, as well as the upper and lower limits of the credibility interval (CI 25%, 50%, 75%, 95%), were reported. The final isotopic space (hereafter referred to as IsoSpace) and the correlation coefficients between prey sources were visually inspected. If the isotopic ratios of two sources were highly overlapped (with an absolute correlation coefficient greater than 0.5) and had similar ecological functions (e.g., occupying the same water layer, similar feeding sources), the sources were merged; otherwise, they were not merged [24,67]. After merging sources, the model was rerun under the same settings (i.e., with the same MCMC parameters). Convergence was assessed using the default MixSIAR diagnostic Gelman–Rubin and Geweke tests. For each fish functional group, the relationship between IsoSpace and the mean contribution of sources to feeding, as a function of covariates, was reported (Table S3).

## 3. Results

### 3.1. Food Web (SIMMr Model)

The Beibu Gulf contains 4 TLs, with the TL range of functional groups, except for primary producers, spanning from the TL of benthic suspension feeders (mainly bivalves) at TL 2 to fish at nearly TL 4. The average TL of fish ranges from 3.28 for planktivorous fish to 3.65 for piscivorous fish (Table 1), but at the species level, the TL of fish ranges from 2.30 to 4.12, spanning 3 TLs.

Phytoplankton is the main food source for copepods (70%), while macro-zooplankton, including juvenile fish and planktonic amphipods, feed on POM, phytoplankton, and copepods in relatively balanced proportions (39%, 30%, and 31%, respectively). Phytoplankton is also the primary food source for benthic suspension feeders (bivalves), making up 90% of their diet. Benthic deposit-feeding animals (such as polychaetes) consume a wide variety of food sources, with a relatively balanced diet, although they also feed on planktivorous fish (32%). Benthic amphipods and omnivorous animals primarily feed on polychaetes and large zooplankton (32% and 21%, respectively). Strongly predatory benthic amphipods and isopods show nearly equal contributions from various food sources, consuming both SOM and polychaetes, as well as a certain proportion of planktivorous and benthic fish (30% and 28%, respectively) (Table 2).

Planktivorous, benthivorous, benthivorous/piscivorous, and planktivorous/piscivorous fish primarily consume zooplankton and fish. Zooplankton comprise 44%, 39%, 43%, and 49% of their diets, respectively. Planktivorous and planktivorous/piscivorous fish mainly feed on copepods, while benthivorous and benthivorous/piscivorous fish primarily consume macro-zooplankton. Benthic invertebrates contribute 13%, 39%, 36%, and 14% to their diet, respectively. Fish contribute 39%, 22%, 14%, and 22%. Additionally, cephalopods contribute 15% to the diet of planktivorous/piscivorous fish. In contrast, piscivorous fish mainly feed on crustaceans (benthic decapoda and stomatopoda), fish, and cephalopods (46%, 28%, and 26%, respectively). Cephalopods primarily feed on crustaceans (71%) and also consume fish (29%) (Table 2).

Overall, pelagic subsidies (i.e., POM, phytoplankton, and zooplankton, excluding pelagic fish) dominate in contribution. In benthic functional groups at lower TLs, these contributions range from 36% to 95%, while in fish functional groups (except piscivorous fish), the contribution ranges from 39% to 49% (Table 2).

### 3.2. Isotope Space in Relation to the Environment

**Figure 2:**
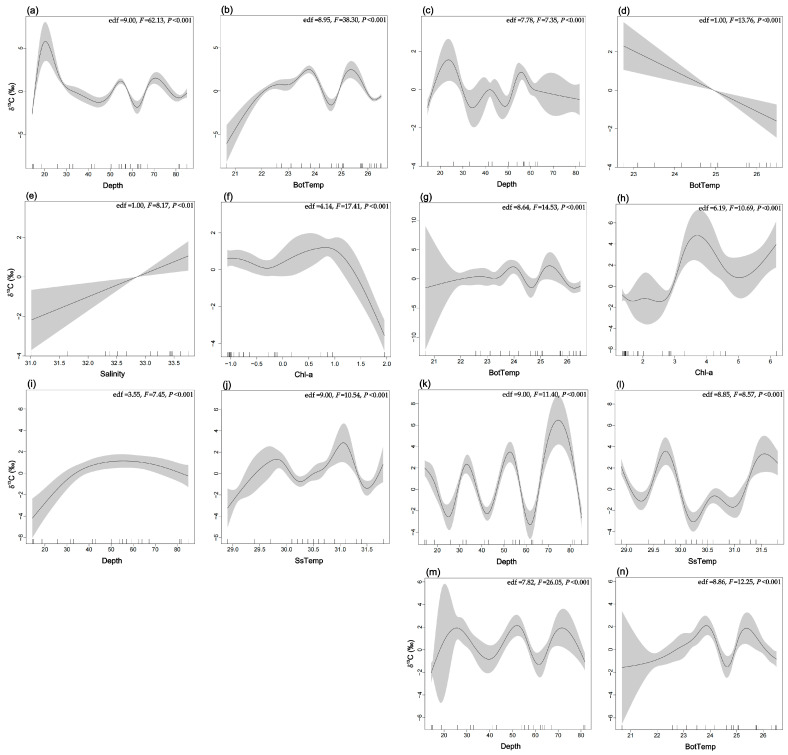
Models relating predicted δ^13^C to the most explanatory environmental parameters for the fish community and functional group models. Solid lines represent the GAM-fitted predictions of the Gaussian distribution. (**a**,**b**) Community scale, (**c**–**e**) planktivorous fish, (**f**,**g**) benthivorous fish, (**h**–**j**) benthivorous/piscivorous fish, (**k**,**l**) planktivorous/piscivorous fish, and (**m**,**n**) piscivorous fish. Shaded areas represent the 95% prediction confidence intervals.

**Figure 3:**
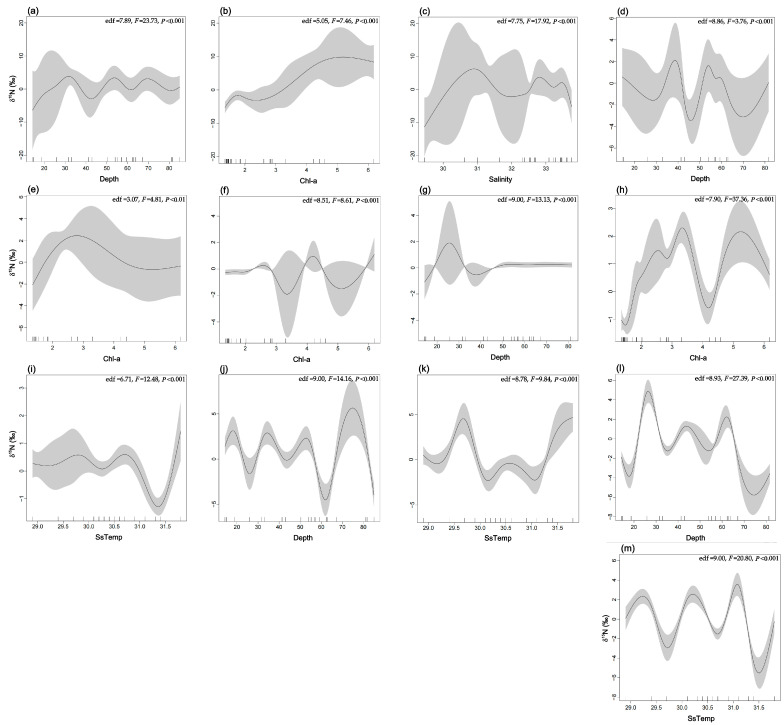
Models relating predicted δ^15^N to the most explanatory environmental parameters for the fish community and functional group models. Solid lines represent the GAM-fitted predictions of the Gaussian distribution. (**a**–**c**) community scale, (**d**,**e**) planktivorous fish, (**f**,**g**) benthivorous fish, (**h**,**i**) benthivorous/piscivorous fish, (**j**,**k**) planktivorous/piscivorous fish, and (**l**,**m**) piscivorous fish. Shaded areas represent the 95% prediction confidence intervals.

At the community scale, models incorporating depth and BotTemp demonstrated the best predictive performance for δ^13^C, explaining 43.7% of the data variance (Table 3). Within the Northern Gulf (Depths < 30 m), δ^13^C was primarily influenced by the depth variable, showing a positive correlation with higher δ^13^C values. In deeper areas (>30 m) of the Southern Gulf, δ^13^C was influenced by both depth and BotTemp. Higher δ^13^C values were observed in the Northern Gulf (around 30 m) and in regions where BotTemps were 22–24 °C and 25–26 °C (Figure 2a,b). For δ^15^N, the best predictive model included depth, Chl–a, and salinity, explaining 57.9% of the variance (Table 3). In areas with high Chl–a (3.0–6.0 mg m^−3^) and low salinity (30.2–31.7), δ^15^N values were higher in the Northern Gulf. In other areas, δ^15^N was influenced by a combination of depth, Chl–a, and salinity, resulting in more complex patterns of variation, with increased uncertainty in the model’s smooth curve estimates (Figure 3a–c). Overall, δ^13^C and δ^15^N exhibited similar spatial patterns, with higher values in the Northern Gulf and more complex variation in other regions driven by multiple variables.

We applied GAMs to δ^13^C and δ^15^N for five fish functional groups with sufficient data. The explained variance in the deviation of δ^13^C values across the functional groups ranged from 47.2% to 59.8% (Table 3), with the best models typically involving 2–3 predictor variables. Depth appeared in all five functional groups, while BotTemp was included in three functional groups. Despite differences in the response curve shapes across the functional groups, planktivorous fish, benthivorous fish, and piscivorous fish showed higher δ^13^C values in the Northern Gulf (<30 m) and Southern Gulf (50–60 m), while benthivorous/piscivorous fish and planktivorous/piscivorous fish showed higher δ^13^C values in the Southern Gulf (>30 m). The influence of other variables on δ^13^C responses across the five fish functional groups was more complex (Figure 2c–n).

For δ^15^N, the models showed good predictive ability, with the explained variance in the deviation ranging from 56.8% to 71.1% (Table 3). The best models included two predictor variables, with depth appearing in four functional groups and both Chl–a and SsTemp in three functional groups. Response curve shapes varied across functional groups. Planktivorous fish exhibited higher δ^15^N values at depths of 30–40 m and 50–60 m, and in areas with Chl–a between 1.0 and 4.0 mg m^−3^. Benthivorous fish had higher δ^15^N values at the Northern Gulf (<30 m). Benthivorous/piscivorous fish showed elevated δ^15^N values in areas with high Chl–a (>1.5 mg m^–3^), while planktivorous/piscivorous fish had higher δ^15^N values at depths >70 m. Piscivorous fish exhibited higher δ^15^N values at depths of 20–30 m, 40–50 m, and 60–70 m (Figure 3d–m).

### 3.3. Fish Diet Variation with Depth (MixSIAR Models)

Based on the results from the GAM fitting, the two most relevant biophysical variables influencing the trophic structure of fish in the Beibu Gulf are depth and Chl–a. Considering that depth and Chl–a exhibit opposite gradient patterns in space, this study uses a previous model (SIMMr) as a template, with the same topology and TEF. The MixSIAR model is then applied to analyze the dietary composition changes in fish functional groups along the depth gradient, while the gradient of Chl–a is incorporated into the discussion.

**Figure 4:**
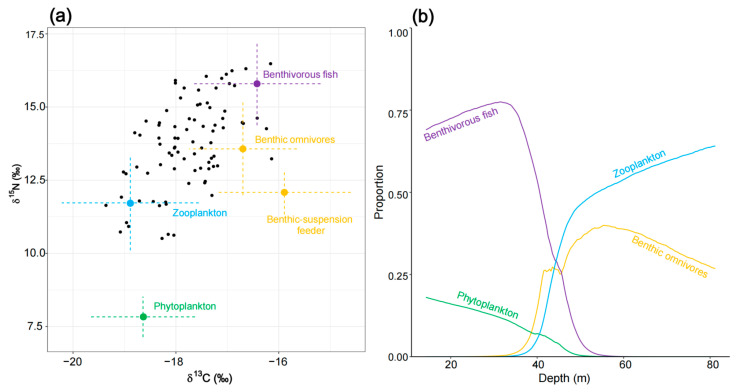
Diet composition of planktivorous fish as estimated by the MixSIAR mixing model. (**a**) Iso–Space displaying consumer’s individual SI values (individual fish black dots) together with sources average SI values. The latter have been adjusted by TEF means and are associated with error bars indicating ±1 SD resulting from combined source and TEF uncertainty. (**b**) Variation in diet as a function of depth is illustrated. Figure 5, Figure 6 and Figure 7 follow the same conventions as this figure.

The initial MixSIAR model for planktivorous fish (N = 84) was run using seven potential food sources. Based on correlation coefficients (copepods and macro-zooplankton had a correlation of –0.64), copepods and macro-zooplankton were merged into a single source labeled “zooplankton”. Similarly, deposit feeders and benthic decapoda/omnivores (with a correlation of −0.64) were combined into the source “benthic omnivores.” This resulted in a five-source mixing model, which was then further analyzed. The results revealed that planktivorous fish primarily rely on benthivorous fish (39.5% ± 20.1%) and the combined source “benthic omnivores” (30.3% ± 21.4%) for their diet, while “zooplankton” served as a secondary food source (16.8% ± 7.9%) (Table S3). When depth was included as a continuous covariate, an inflection point in source contributions was observed at a depth of around 40m. In the shallow Northern Gulf (0–40m), planktivorous fish predominantly consumed benthivorous fish and phytoplankton. As depth increased, the contribution of phytoplankton decreased, while that of benthivorous fish increased. In the deeper Southern Gulf (40–80m), the primary food sources shifted to the combined sources “zooplankton” and “benthic omnivores”. As the depth increased, the contribution of benthic omnivores declined, while that of zooplankton increased (Figure 4).

**Figure 5:**
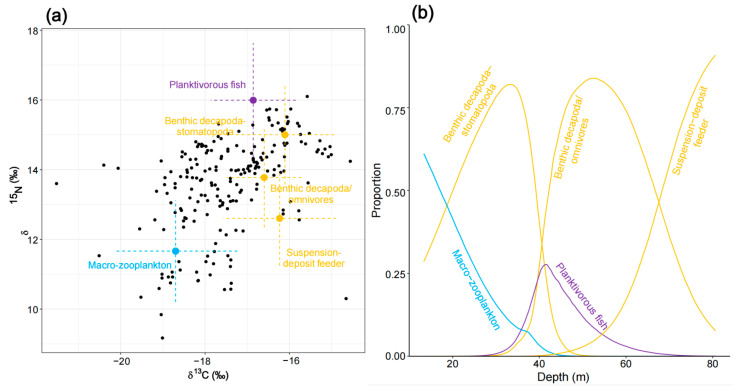
Diet composition of benthivorous fish as estimated by the MixSIAR mixing model. (**a**) Iso–Space and (**b**) variation in diet as a function of depth is illustrated.

The initial MixSIAR model for benthivorous fish (N = 203) was run with eight potential food sources. Based on correlation coefficients (e.g., the correlation between a benthic suspension feeder and deposit feeder was −0.52), the benthic suspension feeder and deposit feeder were combined into a single source, a “suspension–deposit feeder”. Similarly, benthic decapoda/predators and benthic stomatopoda/predators (correlation of −0.52) were merged into the source “benthic decapoda–stomatopoda”, while planktivorous fish and planktivorous/piscivorous fish (correlation of −0.52) were combined into a single source, “planktivorous fish”. This resulted in a five-source mixing model, which was then analyzed further. The results revealed that benthivorous fish primarily feed on benthic decapoda/omnivores (42.2% ± 30.6%) and the combined source “planktivorous fish” (26.9% ± 13.3%). Macro-zooplankton and the combined source “benthic decapoda–stomatopoda” serve as secondary food sources (15.2% ± 20.9% and 14.9% ± 8.1%, respectively) (Table S3). When depth was included as a continuous covariate, a distinct inflection point in the contributions of different sources was observed around depths of 30–40 m. In the shallow Northern Gulf (0–40 m depth range), benthivorous fish mainly feed on macro-zooplankton and the combined source “benthic decapoda–stomatopoda”. As depth increases, the contribution of macro-zooplankton decreases. With further depth, benthivorous fish shift to feeding on benthic decapoda/omnivores, benthic suspension feeders, and piscivorous fish. Notably, along the 20–80 m depth gradient, the contributions of benthic and pelagic resources alternate, but benthic resources remain the primary dietary source (Figure 5).

**Figure 6:**
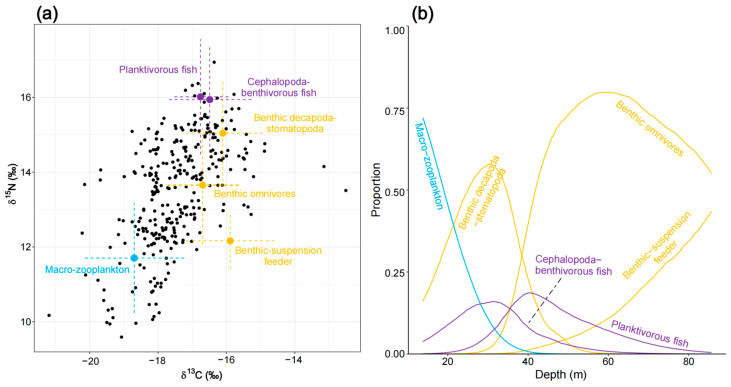
Diet composition of benthivorous/piscivorous fish as estimated by the MixSIAR mixing model. (**a**) Iso–Space and (**b**) variation in diet as a function of depth is illustrated.

The initial MixSIAR model for benthivorous/piscivorous fish (N = 302) was run using nine potential food sources. Based on correlation coefficients (e.g., the deposit feeder and benthic decapoda/omnivores had a correlation of 0.82), deposit feeder and benthic decapoda/omnivores were combined into the source “benthic omnivores”. Similarly, benthic decapoda/predators and benthic stomatopoda/predators (correlation of −0.54) were merged into “benthic decapoda–stomatopoda”, and benthivorous fish and cephalopoda (with a correlation of −0.48) were combined into “cephalopoda–benthivorous fish.” This resulted in a six-source mixing model, which was then further analyzed. The results showed that benthivorous/piscivorous fish primarily feed on the combined source “benthic omnivores” (74.2% ± 10.1%), followed by “benthic decapoda–stomatopoda” (10.0% ± 6.7%) (Table S3). When depth was included as a continuous covariate, a clear inflection point in source contributions was observed at around 40 m. In the shallow Northern Gulf (0–40 m depth range), the contribution of macro-zooplankton rapidly dropped to 0, while the contribution of “benthic decapoda–stomatopoda” increased sharply before declining again. At the same time, the contributions of “benthic omnivores”, “cephalopoda–benthivorous fish”, and piscivorous fish steadily increased. In the deeper Southern Gulf (40–80 m depth range), contributions from fish- and cephalopod-related sources gradually decreased. Across the entire depth gradient, benthic-related functional groups alternated in their contributions, ultimately becoming the primary food sources. However, as depth increased, fishes’ contributions first rose and then declined (Figure 6).

**Figure 7:**
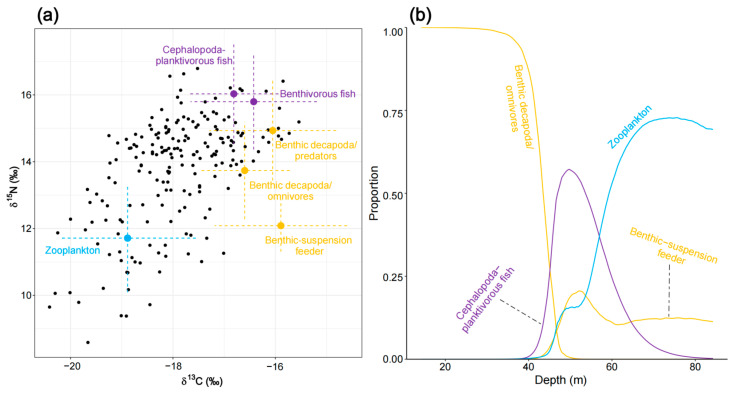
Diet composition of planktivorous/piscivorous fish as estimated by the MixSIAR mixing model. (**a**) Iso–Space and (**b**) variation in diet as a function of depth is illustrated.

The initial MixSIAR model for planktivorous/piscivorous fish (N = 199) was run with eight potential food sources. Based on correlation coefficients (e.g., copepods and macro-zooplankton had a correlation of −0.76), copepods and macro-zooplankton were merged into a single source, “zooplankton”. Similarly, cephalopoda and planktivorous fish (correlation of −0.51) were combined into the source “cephalopoda–planktivorous fish.” This resulted in a six-source mixing model, which was then further analyzed. The results indicated that planktivorous/piscivorous fish primarily feed on the combined source “cephalopoda–planktivorous fish” (42.3% ± 15.6%), followed by benthic decapoda/omnivores (19.2% ± 20.6%), “zooplankton” (15.9% ± 17.5%), and benthic suspension feeder (12.7% ± 12.6%) (Table S3). In the shallow Northern Gulf (0–40 m), planktivorous/piscivorous fish predominantly feed on benthic decapoda/omnivores. In the deeper Southern Gulf (40–80 m), they primarily feed on the combined sources “cephalopoda–planktivorous fish” and “zooplankton,” with a benthic suspension feeder serving as a secondary food source (16.8% ± 7.9%). However, within the 60–80 m depth range, the combined source “zooplankton” replaces “cephalopoda–planktivorous fish” as the primary food source (Figure 7).

**Figure 8:**
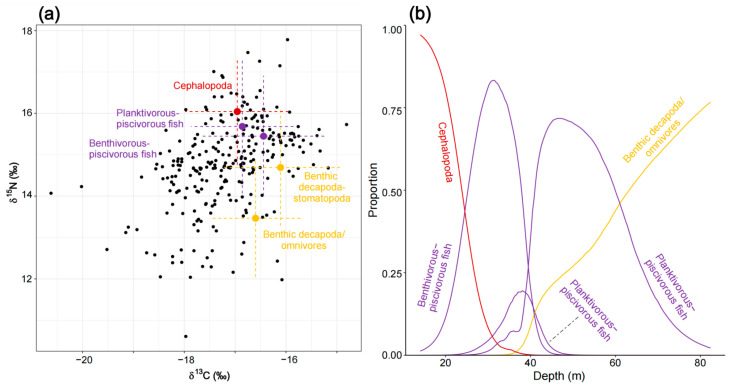
Diet composition of piscivorous fish as estimated by the MixSIAR mixing model. (**a**) Iso–Space and (**b**) variation in diet as a function of depth is illustrated.

The initial MixSIAR model for piscivorous fish (N = 266) was run with eight potential food sources. Based on correlation coefficients (e.g., benthic decapoda/predators and benthic stomatopoda/predators had a correlation of −0.48), benthic decapoda/predators and benthic stomatopoda/predators were combined into the source “benthic decapoda–stomatopoda”. Similarly, planktivorous fish and planktivorous/piscivorous fish (correlation of −0.51) were merged into the source “planktivorous–piscivorous fish”, and benthivorous fish and benthivorous/piscivorous fish (correlation of −0.51) were combined into the source “benthivorous–piscivorous fish”. This resulted in a five-source mixing model, which was then further analyzed. The results revealed that piscivorous fish have relatively evenly distributed contributions from different food sources. They primarily feed on the combined source “benthivorous–piscivorous fish” (30.3% ± 15.9%), “planktivorous–piscivorous fish” (27.0% ± 22.3%), and “benthic decapoda–stomatopoda” (20.2% ± 13.5%), with a secondary contribution from cephalopoda (14.7% ± 21.8%) (Table S3). In the shallow Northern Gulf (0–40 m), as depth increases, the contribution of cephalopoda rapidly declines, while the contribution of “benthivorous–piscivorous fish” increases sharply. After the depth exceeds 40 m, “planktivorous–piscivorous fish” gradually replaces “benthivorous–piscivorous fish”. However, as the depth increases further, benthic decapoda/omnivores gradually take over as the primary food source. Clearly, along the depth gradient, the contribution of swimming functional groups decreases, while the contribution of benthic functional groups increases (Figure 8).

## 4. Discussion

### 4.1. The Trophic Network Structure Associated with Fish in the Beibu Gulf Food Web

The food web of the Beibu Gulf is characterized by four TLs and energy pathways that rely on various carbon sources, including phytoplankton, POM, macroalgae, and SOM. This structure reflects the typical community organization of coastal ecosystems [8,68]. The four TLs include primary producers (trophic level 1), primary consumers (e.g., *Amusium pleuronectes*, trophic level 2.17), and apex predators (e.g., piscivorous *Sphyraena pinguis*, trophic level 4.12). These levels correspond to the trophic classifications by Zhang et al. [28], which categorize organisms into primary producers (marine plants), herbivores (including omnivores), lower-level carnivores (including mesopredators), and higher-level carnivores.

Phytoplankton, POM, and SOM serve as the primary carbon sources within the Beibu Gulf food web, with phytoplankton contributing the largest share. The pelagic carbon sources—particularly phytoplankton and POM—not only support abundant zooplankton populations but also contribute to the deposition of organic matter to the seabed. In the Beibu Gulf, except for the coastal areas with intensive human activities (a water depth of less than 20 m), the sedimentary organic matter in the peripheral regions mainly originates from marine phytoplankton [69]. Therefore, sedimentary organic matter is an important food source for benthic organisms. As such, these carbon sources are critical for both pelagic zooplankton and benthic suspension feeders. This pattern mirrors the significance of phytoplankton in carbon cycling observed in other coastal marine ecosystems [70,71], driven by external nutrient inputs from the summer coastal current in the Beibu Gulf, which supports high levels of primary production, particularly in the Northern Gulf [47]. This highlights that both benthic and pelagic communities in the Beibu Gulf are predominantly fueled by pelagic production, either directly or indirectly, leading to a strong benthic–pelagic coupling effect. These findings align with the conclusions of McMeans et al. [72] and Kopp et al. [8], who emphasized the significant benthic–pelagic coupling in shallow coastal ecosystems.

Although phytoplankton are crucial primary producers in the Beibu Gulf and form the foundation of the marine food web, the contributions of benthic and zooplanktonic organisms (including abundant small fish) to fishes’ diets are equally significant. The trophic interactions involving fish predation also drive a benthic–pelagic coupling effect that should not be overlooked [11]). Planktivorous fish primarily feed on copepods, benthivorous fish, and a small amount of benthic organisms, while benthivorous fish predominantly consume macro-zooplankton, benthic organisms, and some fish. Planktivorous and benthivorous fish include various small fish species (e.g., demersal fish such as *Acropoma japonicum*, *Photopectoralis bindus*, and *Secutor ruconius*, and pelagic fish such as *Bregmaceros mcclellandi*, *Sardinella zunasi*, *Stolephorus commersonnii*). These small fish have high abundance or biomass and are dominant prey in the Northern Gulf, making them crucial food sources for numerous predators (e.g., deposit feeders, benthic decapods/stomatopods/predators, and high-trophic-level fish). They also serve as biological indicators which can be used to identify pelagic fish fishing grounds [28]. Thus, planktivorous and benthivorous fish play key roles in benthic–pelagic coupling in the Beibu Gulf by transferring carbon and energy from both benthic and pelagic sources to higher TLs. Furthermore, planktivorous/piscivorous fish, benthivorous/piscivorous fish, and piscivorous fish—due to their high mobility, ability to switch habitats, and broad predation range—consume a variety of prey, from invertebrates to fish, drawing from both benthic and pelagic sources. In particular, the high vertical migration ability of piscivorous fish significantly intensifies the coupling of pelagic and benthic energy through direct predation.

### 4.2. Biological and Physical Drivers of Fish Feeding Variability (Trophic Variation)

Understanding the biophysical variables that drive the trophic changes in functional groups is crucial for understanding the variability and formation of the food web structure in the Beibu Gulf. Previous studies have indicated that Depth, BotTemp, Chl–a, and Salinity are key factors influencing primary productivity and detritus formation in the Beibu Gulf, which in turn create characteristic environmental gradients. These variables are major drivers in the development of planktonic communities [73,74,75], and they also influence benthic community composition [76,77], particularly the structure of fish communities [26,27,42,78]. Thus, these biophysical processes may further promote spatial reorganization of nutrient networks and trophic functions, contributing to the stability of the food web’s spatial structure.

The results from GAMs indicate that the two most important biophysical variables affecting the trophic structure of fish in the Beibu Gulf are Depth and Chl–a. Additionally, δ^13^C and δ^15^N values of fish communities show consistency along environmental gradients or across different spatial locations. It has been shown that trophic baselines can change spatially along environmental gradients, which in turn influence the stable isotope values of species at higher TLs [55]. Specifically, the δ^13^C and δ^15^N ratios of terrestrial-derived nutrients and detritus differ from marine materials, and their influence gradually decreases along the nearshore–offshore gradient [79]. In the Beibu Gulf, the input of CC results in lower δ^13^C values for primary productivity sources like POM and phytoplankton at the Northern Gulf, reflecting a terrestrial pattern. From the Northern to Southern Gulf, as water depth increases, the influence of coastal currents CC weakens, while the effect of high-temperature, high-salinity SCSW intensifies, leading to a gradient pattern in δ^13^C and δ^15^N values of both benthic and pelagic trophic baselines. Low-trophic-level consumers, such as copepods, euphausiids, mysids, and juvenile fish, are classically considered the ultimate opportunists due to their limited mobility. Consequently [80], they exhibit a passive adaptive feeding strategy and are largely dependent on primary productivity carbon sources [22]. Through the enrichment effects in the food chain, these consumers ultimately influence the stable isotope values of higher-trophic-level fish. Moreover, spatial patterns of δ^13^C and δ^15^N values at the fish community level do not necessarily align with functional groups. Based on the isotope enrichment characteristics within food webs and the differences in δ^13^C values of different basal food sources in the ecosystem, species that primarily feed on pelagic prey tend to have lower δ^13^C values compared to benthic predators [8]. This suggests that different functional groups of fish may exhibit distinct dietary preferences along environmental gradients, driven by their mobility, habitat preferences, and feeding strategies.

### 4.3. Variation in Benthic–Pelagic Coupling Dynamics Mediated by Fish Predation

The movement and foraging patterns of consumers are major drivers of nutrient and energy distribution in ecosystems, playing a key role in maintaining ecosystem stability [81]. Understanding the energy coupling between consumers in coastal marine habitats requires information on spatial resource utilization patterns. The Beibu Gulf spans both tropical and subtropical regions, influenced by three major water masses: the CC, WGCC, and SCSW. The gradual increase in depth from north to south is associated with other oceanic variables, such as primary productivity, salinity, and water temperature. These factors make depth a key variable for explaining habitat variation and its potential impacts on species’ foraging, contributing to a better understanding of the mechanisms underlying the formation of the Beibu Gulf food web structure.

In shallow areas (Northern Gulf), the coupling between benthic and pelagic habitats is strongest, with both planktivorous and benthivorous fish consuming a mix of pelagic and benthic prey. However, as depth increases, the strength of this fish-mediated benthic–pelagic coupling weakens. Planktivorous fish primarily consume pelagic resources, while benthivorous fish focus on benthic resources. This pattern is consistent with studies on food webs in the eastern English Channel [8,24], where the shorter physical distance and weaker physical barriers (such as thermoclines) between benthic and pelagic habitats in shallow waters facilitate stronger benthic–pelagic coupling. Nevertheless, along the depth gradient, planktivorous fish consistently serve as a key coupling agent between benthic and pelagic systems. We hypothesize that this is mainly because planktivorous fish are predominantly small-sized species, which have become dominant prey in the Beibu Gulf food web in recent years, exhibiting high abundance and biomass. These fish also show considerable inter- and intra-species variability in habitat use and foraging patterns [82,83].

**Figure 9:**
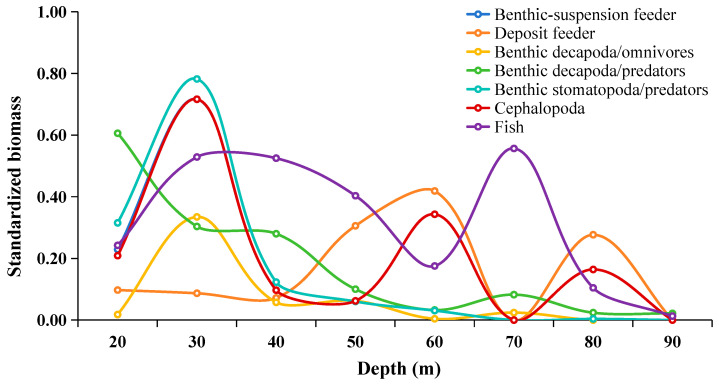
Variation in the biomass of functional groups along the depth gradient in the Beibu Gulf. Biomass data for each functional group were normalized to a 0–1 range to highlight the trend of variation.

As depth increases, the primary foraging targets of fish shift between different functional groups of prey. According to Darnell [84], this adaptive foraging ability, where prey types change along environmental gradients, is one of the key processes for improving population stability and the plasticity of foraging strategies in complex natural communities. It allows fish to cope with local changes in food resource availability and environmental conditions. Moreover, this selectivity and adaptability in foraging might be linked to changes in the trophic environment, such as the composition, abundance, and individual characteristics of consumer and prey communities. However, when we combine the feeding composition of different fish functional groups along the depth gradient (Figure 4, Figure 5, Figure 6, Figure 7 and Figure 8) with the changes in prey resource density (Figure 9), we observe that the trends in these two factors do not reflect a direct influence of prey community composition and abundance on fish resource utilization. Therefore, we speculate that the adaptive foraging mechanism in fish is more complex. Loeuille [29] argues that the main factors influencing consumer adaptive foraging involve both adaptive behaviors and evolutionary aspects. According to optimal foraging theory, fish choose prey or shift from one prey species to another based on the energy balance of foraging activities, while evolution is reflected in the predator’s morphology and physiological traits, which limit the types of prey available [85,86]. The most direct phenotypic trait is body size [87]. Adaptive foraging behaviors are widespread and supported by numerous empirical observations. This suggests that when exploring the reasons for changes in consumers’ resource utilization patterns along environmental gradients, we should not only consider the composition of prey resources but also the biomass and individual characteristics of the available resources (such as the size differences between consumers and prey).

In the depth range of 40–60 m, fish feed from multiple sources, with a clear shift in the contribution of these sources. This area, located in the central part of the Beibu Gulf, is a region where three water masses converge and form upwelling during the summer [88,89], making it one of the most biodiverse and abundant areas in the Gulf. The presence of upwelling likely enhances the fish-mediated coupling between benthic and pelagic systems, allowing fish in this region to occupy a wider ecological niche in terms of foraging. Additionally, the higher fish richness and abundance of available prey in this area may contribute to the differentiation of foraging behaviors both within and between species, which could explain the clear shift in the contribution of prey sources in this depth range.

## 5. Conclusions

This study reveals the trophic network structure associated with fish within the Beibu Gulf food web and explores the dynamic benthic–pelagic coupling mediated by fish across environmental gradients. The GAMs show that depth and Chl–a are the two most significant variables shaping the trophic structure of fish in the Beibu Gulf. Due to their opposing gradient patterns, depth emerges as a key factor for explaining habitat variability and driving adaptive feeding strategies in fish. Bayesian mixing models based on SC and SI data suggest that fish predation plays a pivotal role in linking benthic–pelagic habitats. Planktivorous and benthivorous fish are central to this benthic–pelagic coupling, transferring carbon and energy to higher TLs. In shallow regions (the Northern gulf), the benthic–pelagic coupling effect mediated by fish is most pronounced. As water depth increases, fish exhibit a tendency to feed more within their own habitat (either benthic or pelagic), with prey types constantly shifting, showcasing distinct adaptive feeding behaviors. The findings offer valuable insights into the spatial resource utilization patterns of consumers in the Beibu Gulf, enhancing our understanding of energy coupling dynamics between habitats and providing new perspectives on the formation mechanisms of the Beibu Gulf’s food web structure.

## Figures and Tables

**Table 1:** Functional groups of the food web in the Beibu Gulf. For each functional group, the sample number (N), average δ^13^C and δ^15^N values (± SD), and estimated trophic level (TL ± SD) are listed. The average size of specimens is also provided where available (carapace length for crustaceans, mantle length for cephalopods, and total length for fishes).

Functional Groups	N	Δ^13^C (‰) ± SD	δ^15^N (‰) ± SD	TL ± SD	Size (cm) ± SD
Macroalgae	6	−15.39 ± 1.00	8.26 ± 1.01	1	-
SOM	14	−20.94 ± 2.14	3.21 ± 0.14	1	-
Phytoplankton	4	−19.64 ± 0.93	5.62 ± 0.57	1	-
POM	12	−20.67 ± 2.71	3.58 ± 0.94	1	-
Copepods	14	−20.29 ± 0.81	9.69 ± 1.88	2.17 ± 0.55	-
Macro-zooplankton	30	−19.70 ± 1.41	9.41 ± 1.41	2.09 ± 0.42	-
Benthic suspension feeder	29	−16.89 ± 1.23	9.87 ± 0.64	2.22 ± 0.19	-
Deposit feeder	10	−17.82 ± 1.19	11.16 ± 1.75	2.60 ± 0.51	-
Benthic decapoda/omnivores	12	−17.60 ± 0.78	11.52 ± 1.39	2.71 ± 0.41	3.52 ± 0.52
Benthic decapoda/predators	80	−17.05 ± 1.17	12.72 ± 1.43	3.06 ± 0.42	2.38 ± 2.05
Benthic stomatopoda/predators	25	−17.29 ± 0.81	12.85 ± 1.23	3.10 ± 0.36	2.32 ± 0.54
Planktivorous fish	84	−17.76 ± 0.71	13.72 ± 1.49	3.35 ± 0.44	11.54 ± 3.47
Benthivorous fish	203	−17.42 ± 1.17	13.59 ± 1.36	3.31 ± 0.40	15.75 ± 12.26
Benthivorous/piscivorous fish	302	−17.45 ± 1.1	13.46 ± 1.46	3.28 ± 0.43	15.41 ± 7.11
Planktivorous/piscivorous fish	199	−17.90 ± 1.00	13.75 ± 1.64	3.36 ± 0.48	14.40 ± 3.95
Piscivorous fish	266	−17.13 ± 0.89	14.74 ± 1.08	3.65 ± 0.32	25.18 ± 15.4
Cephalopoda	29	−17.96 ± 0.93	14.10 ± 1.26	3.47 ± 0.37	1.82 ± 0.64

**Table 2:** Diet matrix showing the contribution of each prey (columns) to the diet of consumers (rows) based on SIMMr modeling (10^6^ iterations) in the Beibu Gulf. Each value represents the mean, with the standard deviation in parentheses. Particulate organic matter (POM) and sediment organic matter (SOM) are included.

	SOM	Macroalgae	POM	Phytoplankton	Copepods	Macro-Zooplankton	Benthic-Suspension Feeder	Deposit Feeder	Benthic Decapoda/Omnivores	Benthic Decapoda/Predators	Benthic Stomatopoda/Predators	Planktivorous Fish	Benthivorous Fish	Benthivorous/Piscivorous Fish	Planktivorous/Piscivorous Fish	Piscivorous Fish	Cephalopoda
Copepods			0.30 (0.16)	0.70 (0.16)													
Macro-zooplankton			0.39 (0.11)	0.30 (0.16)	0.31 (0.08)												
Benthic suspension feeder	0.05 (0.04)		0.05 (0.04)	0.90 (0.06)													
Deposit feeder	0.16 (0.10)	0.16 (0.10)	0.15 (0.09)	0.21 (0.14)								0.32 (0.10)					
Benthic decapoda/omnivores	0.09 (0.05)	0.14 (0.08)	0.08 (0.05)	0.13 (0.09)		0.21 (0.14)		0.35 (0.15)									
Benthic decapoda/predators	0.18 (0.03)	0.06 (0.04)					0.10 (0.07)	0.18 (0.15)	0.18 (0.14)			0.15 (0.10)	0.15 (0.11)				
Benthic stomatopoda/predators	0.21 (0.04)						0.09 (0.06)	0.15 (0.12)	0.15 (0.12)	0.12 (0.09)		0.15 (0.10)	0.13 (0.09)				
Planktivorous fish				0.04 (0.02)	0.37 (0.06)	0.07 (0.06)	0.03 (0.02)	0.05 (0.04)	0.05 (0.04)				0.39 (0.05)				
Benthivorous fish						0.39 (0.04)	0.05 (0.03)	0.09 (0.07)	0.08 (0.06)	0.08 (0.06)	0.09 (0.07)	0.11 (0.07)			0.11 (0.07)		
Benthivorous/piscivorous fish						0.43 (0.03)	0.05 (0.03)	0.08 (0.07)	0.08 (0.06)	0.07 (0.05)	0.08 (0.06)	0.07 (0.05)	0.07 (0.05)				0.07 (0.04)
Planktivorous/piscivorous fish					0.39 (0.08)	0.10 (0.08)	0.03 (0.02)		0.05 (0.04)	0.06 (0.04)		0.13 (0.09)	0.09 (0.07)				0.15 (0.09)
Piscivorous fish									0.42 (0.04)	0.02 (0.01)	0.02 (0.02)	0.05 (0.05)	0.02 (0.02)	0.02 (0.02)	0.19 (0.16)		0.26 (0.15)
Cephalopoda									0.59 (0.12)	0.06 (0.05)	0.06 (0.06)	0.06 (0.06)	0.06 (0.05)	0.06 (0.05)	0.07 (0.06)	0.04 (0.03)	

**Table 3:** Summary of the Generalized Additive Model (GAM) relationships between the most explanatory environmental variables and δ^13^C and δ^15^N in the Beibu Gulf of community-level and functional group models. The environmental variables include depth, SsTemp, BotTemp, Chl–a, and salinity.

	Model Formula	N. Obs	AIC	GCV	%dev	*r^2^*
Carbon (δ^13^C)						
Community scale	δ^13^C~Depth + BotTemp	1054	2459.63	0.60	0.474	0.465
Planktivorous fish	δ^13^C~Depth + BotTemp + Salinity	84	150.77	0.33	0.495	0.428
Benthivorous fish	δ^13^C~Chl–a + BotTemp	203	502.24	0.68	0.598	0.562
Benthivorous/piscivorous fish	δ^13^C~Chl–a + Depth + SsTemp	302	697.56	0.58	0.576	0.547
Planktivorous/piscivorous fish	δ^13^C~Depth + SsTemp	199	480.88	0.64	0.472	0.420
Piscivorous fish	δ^13^C~Depth + BotTemp	266	495.88	0.37	0.592	0.565
Nitrogen (δ^15^N)						
Community scale	δ^15^N~Depth + Chl–a + Salinity	1054	2954.98	0.96	0.579	0.570
Planktivorous fish	δ^15^N~Depth + Chl–a	84	234.31	0.89	0.711	0.662
Benthivorous fish	δ^15^N~Depth + Chl–a	203	525.92	0.76	0.658	0.626
Benthivorous/piscivorous fish	δ^15^N~Chl–a + SsTemp	302	811.61	0.85	0.640	0.621
Planktivorous/piscivorous fish	δ^15^N~Depth + SsTemp	199	610.85	1.23	0.622	0.585
Piscivorous fish	δ^15^N~Depth + SsTemp	266	615.32	0.58	0.568	0.537

## Data Availability

Data are available upon request from the corresponding author; they are not publicly available due to ongoing comparative analyses.

## Supplementary Materials

The following supporting information can be downloaded at https://www.mdpi.com/article/10.3390/biology14020207/s1, Figure S1: Kriging interpolation of trophic baseline isotope ratios of *Aequipecten opercularis*; Table S1: Feeding statistics of fish in the Beibu Gulf; Table S2: Summary of SIMMr analysis results of food source contribution of fish functional groups; Table S3: Summary of MixSIAR analysis results of food source contribution of fish functional groups.

## Appendix A

**Table A1:** Trophic ecology of primary carbon sources, invertebrate, and fish species in this study. Includes functional groups, species count, species composition description, functional group characteristics, and references.

Functional Groups	No. of Species	Species Composition Description	Functional Group Characteristics	Reference
SOM	1	-	-	-
Macroalgae	1	-	-	-
POM	1	-	-	-
Phytoplankton	1	-	-	-
Copepods	1	Copepods	Feed on particulate organic matter and phytoplankton	[90]
Macro-zooplankton	4	Fish larvae, Sergestidae, Planktonic amphipods, Crustacean larvae	Feed on copepods and other zooplankton	[91]
Benthic suspension feeder	2	Bivalves and gastropods *Amusium pleuronectes*, *Scapharca kagoshimensis*, *Turritella terebra*	Feed on organic particles, phytoplankton, zooplankton, and bacteria	[57]
Deposit feeder	1	Polychaeta	Omnivorous animals that feed on phytoplankton, zooplankton, crustaceans, and other worms	[92,93]
Benthic Decapoda/omnivores	2	Shrimps. Decapoda *Metapenaeopsis barbata*, *Solenocera crassicornis*	Omnivorous animals that feed on algae, crustaceans, gastropods, and polychaetes	[52,94]
Benthic Decapoda/predators	20	Shrimp *Metapenaeus joyneri*, *Melicertus marginatus*, and crabs *Portunus pelagicus*, *Charybdis orientalis*, etc.	Carnivorous/scavenger animals that feed on crustaceans, marine worms, mollusks, and dead fish	[95,96]
Benthic Stomatopoda/predators	7	Stomatopoda *Harpiosquilla harpax*, *Oratosquilla manningi*, and *Harpiosquilla annandalei*, etc.	Predatory animals that feed on crustaceans, small fish, mollusks, or other small invertebrates	[96]
Planktivorous fish	10	Small fish *Acropoma japonicum*, *Thryssa vitrirostris*, *Sardinella zunasi*, *Stolephorus commersonnii*, *Thryssa dussumieri*, and *Bregmaceros mcclellandi*; medium and small–sized fish *Alepes djedaba*, *Pampus argenteus*, *Pampus chinensis*, and *Polydactylus sextarius*	Primarily feed on plankton; this type is mostly composed of pelagic fish. Their upper and lower jaw teeth are small or degenerated, and their gill rakers are fine and well developed, allowing them to filter small planktonic organisms [28]	Table S1
Benthivorous fish	36	Osteichthyes/Perciformes *Telatrygon zugei*; Rajiformes *Okamejei boesemani*; Osteichthyes/ Pleuronectiformes *Brachypleura novaezeelandiae*, *Cynoglossus nanhaiensis*, Anguilliformes *Pisodonophis boro* and *Pisodonophis cancrivorus*; Tetraodontiformes *Thamnaconus hypargyreus*; Scorpaeniformes *Lepidotrigla japonica*; demersal fish Perciformes *Gerres japonicus* and *Nemipterus japonicus*, etc.	Primarily feed on benthic organisms; this type is mostly composed of bottom-dwelling or benthic fish. Due to the complex composition of their diet, the morphology of their jaw teeth is more varied. As they consume foods of different sizes, their stomachs are also quite diverse in shape.	Table S1
Benthivorous/piscivorous fish	27	Anguilliformes *Dysomma anguillare*, *Gnathophis heterognathos*, *Gymnothorax cribroris*, *Scolecenchelys macropterus*; *Siluriformes Plicofollis nella*; Tetraodontiformes *Lagocephalus spadiceus*; Scorpaeniformes *Grammoplites scaber*, *Neomerinthe procurva*, *Onigocia* macrolepis, etc.; Ophidiiformes *Brotula multibarbata*, *Sirembo imberbis*; Perciformes *Champsodon atridorsalis*, *Epinephelus coioides*, *Epinephelus latifasciatus*, etc.	Fish that primarily feed on benthic organisms and swimmers animals that belong to this type. These fish are mostly benthic species, inhabiting shallow seabed areas, and are generally more aggressive and voracious.	Table S1
Planktivorous/piscivorous fish	17	Clupeiformes *Setipinna breviceps*, *Thryssa hamiltoni*; Syngnathiformes *Fistularia commersonii*; Perciformes *Alepes kleinii*, *Alepes melanoptera*, *Atropus atropos*, *Atule mate*, etc.	Most of these fish are pelagic species that tend to migrate in schools. In their juvenile stage, they primarily feed on zooplankton, while as mature, diet shifts to larger zooplankton, small fish, and some benthic organisms.	Table S1
Piscivorous fish	26	Chondrichthyes/Carcharhiniformes *Scoliodon laticaudus*; Osteichthyes/Lophiiformes *Lophiomus setigerus*; Clupeiformes *Chirocentrus nudus*, *Ilisha melastoma*, *Paracentropogon rubripinnis*; Anguilliformes *Gymnothorax reevesii*, *Gymnothorax reticularis*, *Muraenesox cinereus*, *Rhynchoconger ectenurus*; Aulopiformes *Saurida tumbil*, *Saurida undosquamis*; Perciformes *Branchiostegus albus*, *Branchiostegus argentatus*, *Pennahia anea*, *Pennahia pawak*, etc.	Primary diet consists of fish and cephalopods. This group includes both benthic and pelagic species. These fish are aggressive, voracious, and have strong swimming abilities. They have large mouths with powerful, sharp teeth on both the upper and lower jaws, often featuring inward-facing or backward-curved canine teeth to prevent prey from escaping. Their gill rakers are sparse, short, or degenerated [28].	Table S1
Cephalopoda	4	*Loligo chinensis*, *Sepioteuthis lessoniana*, *Sepia brevimana*, and *Sepiella maindroni*	Primarily prey on invertebrates and fish	[97,98]
