# Supplementary material for: Fish Community Resource Utilization Reveals Benthic–Pelagic Trophic Coupling Along Depth Gradients in the Beibu Gulf, South China Sea
Source: Biology (Basel). 2025 Feb 16;14(2):207. doi: 10.3390/biology14020207 (PMC11851788; doi:10.3390/biology14020207)
Supplement: Supplementary file 1 [file biology-14-00207-s001.zip › biology-3442789-supplementary.pdf]

Supplementary materials

Article

Fish Community Resource Utilization Reveals Benthic–Pelagic Trophic Coupling along Depth Gradients in the Beibu Gulf, South China Sea

Xiaodong Yang [1], Konglan Luo [1], Jiawei Fu [1],Bin Kang [2], Xiongbo He [1,3,*] and Yunrong Yan [1,3,*]

1 Fisheries College, Guangdong Ocean University, Zhanjiang 524088, China; yangxd2832@163.com (X.Y.); luokonlan@gmail.com (K.L.); fjw18884902029@outlook.com (J.F.)
2 Fisheries College, Ocean University of China, Qingdao 266003, China; kangbin@ouc.edu.cn
3 Guangdong Provincial Engineering and Technology Research Center of Far Sea Fisheries Management and Fishing of South China Sea, Guangdong Ocean University, Zhanjiang 524088, China
* Correspondence: xiongbo98@163.com (X.H.); yryan_gdou@163.com (Y.Y.); Tel.: +86–13729006487 (X.H.); +86–13902505881 (Y.Y.)

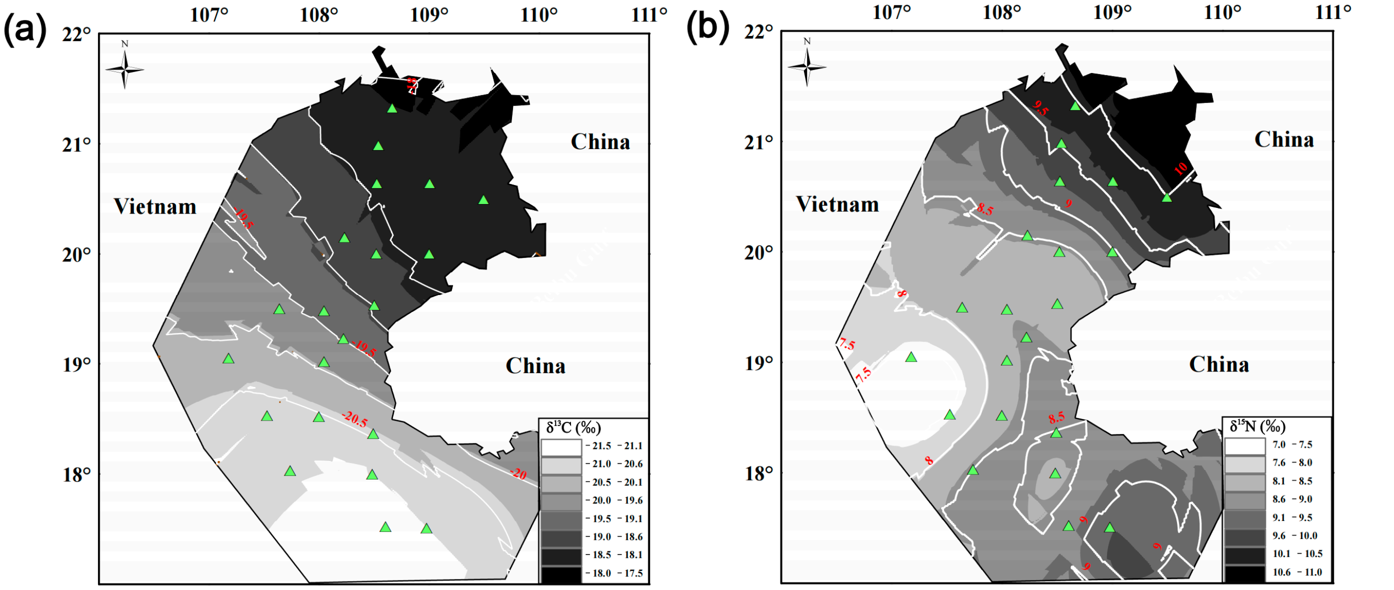

Figure S1. Kriging interpolation of trophic baseline isotope ratios of *Aequipecten opercularis*. (**a**) Mean $\delta^{15}N$ and (**b**) Mean $\delta^{13}C$. Green triangles represent the sampling locations of *A. opercularis*, and the white solid lines are the isoclines of isotope ratios.

Table·S1. Feeding statistics of fish in the Beibu Gulf. Includes functional groups, species composition, number of samples (excluding empty stomachs), and diet composition. "Swimming animals" includes fish and cephalopods. Total samples were collected from the Beibu Gulf (2008-2022).

Functional groups	Species	No.of samples	Diet composition%		
			Plankton	Benthos	Swimming animals
Planktivorousfish	*Polydactylus sextarius*	96	12.5	45.8	41.7
	Acropoma japonicum	52	19.2	32.7	48.1
	Thryssa dussumieri	43	11.6	44.2	44.2
	Sardinella zunasi	23	17.4	21.7	60.9
	Pampus argenteus	20	30.0	20.0	50.0
	Pampus chinensis	14	57.1	42.9	0.0
	Alepes djedaba	7	57.1	0.0	42.9
	Bregmaceros mcclellandi	5	60.0	20.0	20.0
	Stolephorus commersonnii	4	25.0	75.0	0.0
	Thryssa vitrirostris	3	100.0	0.0	0.0
Benthivorousfish	*Nemipterus japonicus*	325	0.0	71.7	28.3
	Upeneus sulphureus	169	0.0	69.8	30.2
	Nemipterus marginatus	142	0.0	66.2	33.8
	Nemipterus bathybius	116	0.0	52.6	47.4
	Brachypleura novaezeelandiae	89	0.0	60.7	39.3
	Poecilopsetta plinthus	88	0.0	60.2	39.8
	Nemipterus nemurus	39	0.0	59.0	41.0
	Okamejei boesemani	33	3.0	60.6	36.4
	Telatrygon zugei	25	0.0	72.0	28.0
	Neolaeops microphthalmus	18	0.0	66.7	33.3
	Parachaeturichthys polynema	17	0.0	70.6	29.4
	Hapalogenys analis	15	0.0	66.7	33.3
	Sillago japonica	14	0.0	71.4	28.6

	Zebrias zebra	11	0.0	81.8	18.2
	Pseudorhombus cinnamoneus	11	0.0	63.6	36.4
	Leiognathus berbis	11	0.0	81.8	18.2
	Gobiopsis macrostoma	10	0.0	60.0	40.0
	Secutor ruconius	10	0.0	60.0	40.0
	Lepidotrigla japonica	10	0.0	70.0	30.0
	Trypauchen vagina	9	0.0	66.7	33.3
	Oxyurichthys auchenolepis	9	0.0	66.7	33.3
	Photopectoralis bindus	9	0.0	77.8	22.2
	Upeneus luzonius	8	0.0	62.5	37.5
	Myersina filifer	8	0.0	75.0	25.0
	Pisodonophis cancrivorus	7	0.0	71.4	28.6
	Cynoglossus nanhaiensis	7	0.0	71.4	28.6
	Cynoglossus puncticeps	7	0.0	85.7	14.3
	Thamnaconus hypargyreus	7	0.0	100.0	0.0
	Pseudorhombus arsius	6	0.0	83.3	16.7
	Gerres japonicus	5	0.0	60.0	40.0
	Cynoglossus oligolepis	5	0.0	100.0	0.0
	Callionymus curvicornis	5	0.0	80.0	20.0
	Pisodonophis boro	5	0.0	100.0	0.0
	Acentrogobius caninus	4	0.0	50.0	50.0
	Leiognathus brevirostris	4	0.0	75.0	25.0
	Solea ovata	4	0.0	75.0	25.0
Benthivorous/piscivorousfish	*Jaydia poecilopterus*	152	2.6	48.0	49.3
	Terapon theraps	133	0.8	32.3	66.9
	Evynnis cardinalis	117	0.0	29.9	70.1
	Johnius belangerii	112	0.9	31.3	67.9
	Dysomma anguillare	60	1.7	43.3	55.0
	Priacanthus macracanthus	40	0.0	32.5	67.5
	Parapercis ommatura	36	0.0	36.1	63.9
	Sirembo imberbis	32	0.0	43.8	56.3
	Lagocephalus spadiceus	31	3.2	35.5	61.3
	Jaydia striata	29	6.9	31.0	62.1
	Grammoplites scaber	24	0.0	54.2	45.8
	Jaydia truncata	23	4.3	39.1	56.5
	Pennahia macrocephalus	18	0.0	33.3	66.7
	Gnathophis heterognathos	13	0.0	38.5	61.5
	Plicofollis nella	13	0.0	53.8	46.2
	Scorpaenopsis neglecta	12	0.0	50.0	50.0
	Champsodon atridorsalis	11	0.0	36.4	63.6
	Selaroides leptolepis	11	0.0	54.5	45.5
	Epinephelus latifasciatus	11	0.0	63.6	36.4
	Brotula multibarbata	10	0.0	40.0	60.0
	Neomerinthe procurva	9	0.0	55.6	44.4
	Gymnothorax cribroris	9	0.0	44.4	55.6
	Pterois lunulata	8	0.0	62.5	37.5

	Thysanophrys chiltonae	7	0.0	42.9	57.1
	scolecenchelys macropterus	7	0.0	71.4	28.6
	Epinephelus coioides	5	0.0	60.0	40.0
	Onigocia macrolepis	4	0.0	50.0	50.0
Planktivorous/piscivorousfish	*Decapterus maruadsi*	273	12.5	13.2	74.4
	Lactarius lactarius	87	2.3	17.2	80.5
	Alepes kleinii	64	1.6	43.8	54.7
	Mene maculata	45	8.9	22.2	68.9
	Ostorhinchus pleuron	44	4.5	40.9	54.5
	Setipinna breviceps	43	4.7	41.9	53.5
	Trachurus japonicus	40	15.0	25.0	60.0
	Atule mate	27	48.1	14.8	37.0
	Psenopsis anomala	26	19.2	19.2	61.5
	Siganus fuscescens	22	22.7	31.8	45.5
	Cubiceps whiteleggii	18	44.4	11.1	44.4
	Parastromateus niger	13	15.4	7.7	76.9
	Fistularia commersonii	12	16.7	0.0	83.3
	Thryssa hamiltoni	11	36.4	0.0	63.6
	Atropus atropos	10	60.0	20.0	20.0
	Alepes melanoptera	7	42.9	14.3	42.9
	Ostorhinchus semilineatus	4	25.0	25.0	50.0
Piscivorousfish	*Saurida tumbil*	2745	0.0	7.0	93.0
	Pennahia pawak	1975	0.1	35.1	64.9
	Pennahia anea	1496	0.1	30.5	69.4
	Saurida undosquamis	389	0.0	6.4	93.6
	Trichiurus brevis	129	0.0	7.8	92.2
	Trichiurus japonicus	101	0.0	7.9	92.1
	Scoliodon laticaudus	88	0.0	35.2	64.8
	Terapon jarbua	70	1.4	25.7	72.9
	Muraenesox cinereus	65	1.5	33.8	64.6
	Carangoides malabaricus	50	0.0	8.0	92.0
	Lophiomus setigerus	38	0.0	13.2	86.8
	Branchiostegus albus	37	0.0	24.3	75.7
	Branchiostegus argentatus	20	0.0	15.0	85.0
	Rhynchoconger ectenurus	19	0.0	15.8	84.2
	Chirocentrus nudus	18	0.0	0.0	100.0
	Rastrelliger kanagurta	13	0.0	7.7	92.3
	Uranoscopus tosae	13	0.0	15.4	84.6
	Trichiurus nanhaiensis	11	0.0	0.0	100.0
	Sphyraena barracuda	11	0.0	0.0	100.0
	Gymnothorax reticularis	10	0.0	30.0	70.0
	Dendrochirus zebra	10	0.0	20.0	80.0
	Scomberomorus commerson	6	0.0	0.0	100.0
	Paracentropogon rubripinnis	6	0.0	0.0	100.0
	Sphyraena pinguis	6	0.0	0.0	100.0
	Gymnothorax reevesii	5	0.0	20.0	80.0

Ilisha melastoma	4	0.0	0.0	100.0

Table·S2. Summary of SIMMr analysis results of food source contribution of fish functional groups

Functionalgroups	Foodsource	Mean	SD	2.5%	25%	50%	75%	97.5%
Copepods	POM	0.30	0.16	0.05	0.17	0.28	0.41	0.62
	Phytoplankton	0.70	0.16	0.38	0.59	0.72	0.83	0.95
Macro-zooplankton	POM	0.39	0.11	0.15	0.33	0.40	0.47	0.57
	Phytoplankton	0.30	0.16	0.06	0.18	0.28	0.40	0.67
	Copepods	0.31	0.08	0.14	0.26	0.31	0.36	0.44
Benthic-suspension feeder	BOM	0.05	0.04	0.01	0.02	0.04	0.06	0.14
	POM	0.05	0.04	0.01	0.02	0.04	0.07	0.16
	Phytoplankton	0.90	0.06	0.74	0.87	0.92	0.95	0.98
Deposit feeder	BOM	0.16	0.10	0.02	0.08	0.14	0.22	0.38
	POM	0.15	0.09	0.02	0.08	0.13	0.21	0.38
	Phytoplankton	0.21	0.14	0.02	0.10	0.19	0.30	0.54
	Macroalgae	0.16	0.10	0.02	0.08	0.14	0.21	0.39
	Planktivorous fish	0.32	0.10	0.10	0.26	0.33	0.39	0.51
Benthic decapoda/omnivores	BOM	0.09	0.05	0.01	0.04	0.07	0.12	0.22
	POM	0.08	0.05	0.01	0.04	0.07	0.11	0.20
	Phytoplankton	0.13	0.09	0.02	0.06	0.11	0.18	0.34
	Macroalgae	0.14	0.08	0.02	0.08	0.13	0.19	0.33
	Macro-zooplankton	0.21	0.14	0.02	0.09	0.18	0.31	0.53
	Deposit feeder	0.35	0.15	0.06	0.25	0.37	0.47	0.61
Benthic decapoda/predators	BOM	0.18	0.03	0.11	0.15	0.18	0.20	0.24
	Macroalgae	0.07	0.04	0.01	0.04	0.06	0.09	0.15
	Benthic-suspension feeder	0.11	0.07	0.01	0.05	0.09	0.15	0.29
	Deposit feeder	0.18	0.15	0.02	0.07	0.14	0.26	0.54
	Benthic decapoda/omnivores	0.18	0.14	0.02	0.07	0.13	0.25	0.54
	Planktivorous fish	0.15	0.10	0.02	0.07	0.13	0.21	0.37
	Benthivorous fish	0.15	0.11	0.02	0.06	0.12	0.21	0.42
Benthic stomatopoda/predators	BOM	0.20	0.04	0.13	0.18	0.20	0.23	0.27
	Benthic-suspension feeder	0.09	0.06	0.01	0.04	0.08	0.13	0.25
	Deposit feeder	0.15	0.12	0.02	0.06	0.12	0.21	0.45
	Benthic decapoda/omnivores	0.15	0.12	0.02	0.06	0.12	0.22	0.46
	Benthic decapoda/predators	0.12	0.09	0.01	0.05	0.10	0.17	0.36
	Planktivorous fish	0.15	0.10	0.02	0.07	0.13	0.21	0.40
	Benthivorous fish	0.13	0.09	0.01	0.06	0.11	0.18	0.36
Planktivorous fish	Phytoplankton	0.02	0.02	0.00	0.01	0.02	0.03	0.06
	Macroalgae	0.02	0.01	0.00	0.01	0.02	0.02	0.04
	Copepods	0.37	0.06	0.22	0.34	0.38	0.42	0.47
	Macro-zooplankton	0.07	0.06	0.01	0.03	0.05	0.09	0.23
	Benthic-suspension feeder	0.03	0.02	0.00	0.01	0.02	0.04	0.07
	Deposit feeder	0.05	0.04	0.01	0.02	0.04	0.06	0.15
	Benthic decapoda/omnivores	0.05	0.04	0.01	0.02	0.04	0.07	0.16
	Benthivorous fish	0.39	0.05	0.28	0.36	0.39	0.43	0.48
Benthivorous fish	Macro-zooplankton	0.39	0.04	0.31	0.36	0.39	0.41	0.45
	Benthic-suspension feeder	0.05	0.03	0.01	0.03	0.04	0.07	0.12
	Deposit feeder	0.09	0.07	0.01	0.04	0.07	0.12	0.26
	Benthic decapoda/omnivores	0.08	0.06	0.01	0.04	0.07	0.11	0.25
	Benthic decapoda/predators	0.08	0.06	0.01	0.03	0.06	0.10	0.22
	Benthic stomatopoda/predators	0.09	0.07	0.01	0.04	0.07	0.12	0.28
	Planktivorous fish	0.11	0.07	0.01	0.05	0.10	0.16	0.28
	Planktivorous/piscivorous fish	0.12	0.07	0.01	0.06	0.11	0.16	0.27
Benthivorous/piscivorous fish	Macro-zooplankton	0.43	0.03	0.36	0.41	0.43	0.45	0.48
	Benthic-suspension feeder	0.05	0.03	0.01	0.03	0.05	0.07	0.13
	Deposit feeder	0.08	0.07	0.01	0.04	0.07	0.11	0.26
	Benthic decapoda/omnivores	0.08	0.06	0.01	0.03	0.06	0.11	0.24
	Benthic decapoda/predators	0.07	0.05	0.01	0.03	0.06	0.10	0.21
	Benthic stomatopoda/predators	0.08	0.06	0.01	0.03	0.06	0.10	0.23
	Planktivorous fish	0.07	0.05	0.01	0.03	0.06	0.10	0.20
	Benthivorous fish	0.07	0.05	0.01	0.03	0.06	0.10	0.20
	Cephalopoda	0.07	0.04	0.01	0.03	0.06	0.09	0.17
Planktivorous/piscivorous fish	Copepods	0.39	0.08	0.17	0.35	0.41	0.44	0.49
	Macro-zooplankton	0.10	0.08	0.01	0.04	0.07	0.13	0.34
	Benthic-suspension feeder	0.03	0.02	0.01	0.02	0.03	0.05	0.09
	Benthic decapoda/omnivores	0.05	0.04	0.01	0.03	0.04	0.07	0.15

	Benthic decapoda/predators	0.06	0.04	0.01	0.03	0.05	0.07	0.16
	Planktivorous fish	0.13	0.09	0.01	0.05	0.11	0.19	0.32
	Benthivorous fish	0.09	0.07	0.01	0.04	0.07	0.12	0.27
	Cephalopoda	0.16	0.09	0.02	0.08	0.15	0.23	0.33
Piscivorous fish	Benthic decapoda/omnivores	0.42	0.04	0.34	0.39	0.42	0.44	0.49
	Benthic decapoda/predators	0.02	0.01	0.00	0.01	0.01	0.02	0.05
	Benthic stomatopoda/predators	0.02	0.02	0.00	0.01	0.02	0.03	0.07
	Planktivorous fish	0.05	0.05	0.01	0.02	0.03	0.06	0.17
	Benthivorous fish	0.02	0.02	0.00	0.01	0.02	0.03	0.07
	Benthivorous/piscivorous fish	0.02	0.02	0.00	0.01	0.02	0.03	0.07
	Planktivorous/piscivorous fish	0.19	0.16	0.01	0.05	0.14	0.33	0.51
	Cephalopoda	0.26	0.15	0.02	0.12	0.29	0.39	0.47
Cephalopoda	Benthic decapoda/omnivores	0.59	0.12	0.32	0.52	0.60	0.67	0.79
	Benthic decapoda/predators	0.06	0.05	0.01	0.02	0.04	0.08	0.19
	Benthic stomatopoda/predators	0.06	0.06	0.01	0.02	0.04	0.08	0.22
	Planktivorous fish	0.06	0.06	0.01	0.03	0.05	0.08	0.22
	Benthivorous fish	0.06	0.05	0.01	0.02	0.04	0.07	0.19
	Benthivorous/piscivorous fish	0.06	0.05	0.01	0.02	0.05	0.08	0.18
	Planktivorous/piscivorous fish	0.07	0.06	0.01	0.03	0.05	0.10	0.24
	Piscivorous fish	0.04	0.03	0.01	0.02	0.03	0.05	0.12

Table·S3. Summary of MixSIAR analysis results of food source contribution of fish functional groups.

Functional groups	Food source	Mean	SD	2.5%	25%	50%	75%	97.5%
Planktivorous fish	Benthic-suspensionfeeder	0.057	0.039	0.014	0.028	0.042	0.077	0.163
	Benthic omnivores	0.303	0.214	0.017	0.132	0.256	0.435	0.789
	Benthivorous fish	0.395	0.206	0.043	0.217	0.397	0.585	0.746
	Phytoplankton	0.077	0.056	0.017	0.034	0.060	0.108	0.243
	Zooplankton	0.168	0.079	0.033	0.111	0.156	0.208	0.336
Benthivorous fish	Benthic decapoda-stomatopoda	0.149	0.081	0.029	0.091	0.137	0.194	0.332
	Benthic decapoda/omnivores	0.422	0.306	0.000	0.007	0.547	0.673	0.803
	Macro-zooplankton	0.152	0.209	0.001	0.004	0.010	0.382	0.552
	Planktivorous fish	0.269	0.133	0.074	0.153	0.256	0.381	0.515
	Suspension-deposit feeder	0.008	0.007	0.000	0.002	0.005	0.012	0.025
Benthivorous/piscivorous fish	Benthic-suspension feeder	0.046	0.047	0.001	0.011	0.031	0.063	0.206
	Benthic decapoda-stomatopoda	0.100	0.067	0.006	0.024	0.109	0.148	0.222
	Benthic omnivores	0.742	0.101	0.532	0.681	0.737	0.791	0.944
	Cephalopoda-benthivorous fish	0.071	0.058	0.005	0.025	0.053	0.106	0.209
	Macro-zooplankton	0.001	0.001	0.000	0.000	0.001	0.002	0.004
	Planktivorous fish	0.040	0.036	0.004	0.015	0.032	0.047	0.152
Planktivorous/piscivorous fish	Benthic-suspension feeder	0.127	0.126	0.005	0.017	0.069	0.250	0.387
	Benthic decapoda/omnivores	0.192	0.206	0.001	0.008	0.143	0.305	0.705
	Benthic decapoda/predators	0.052	0.065	0.003	0.013	0.027	0.065	0.217
	Benthivorous fish	0.048	0.049	0.001	0.009	0.032	0.069	0.188
	Cephalopoda-planktivorous fish	0.423	0.156	0.123	0.303	0.430	0.547	0.680
	Zooplankton	0.159	0.175	0.010	0.026	0.070	0.274	0.535
Piscivorous fish	Benthic decapoda-stomatopoda	0.202	0.135	0.025	0.096	0.181	0.284	0.505
	Benthic decapoda/omnivores	0.078	0.065	0.002	0.018	0.066	0.112	0.237
	Benthivorous-piscivorous fish	0.303	0.159	0.067	0.168	0.275	0.428	0.616
	Cephalopoda	0.147	0.218	0.000	0.001	0.002	0.334	0.608
	Planktivorous-piscivorous fish	0.270	0.223	0.001	0.017	0.260	0.441	0.700
